# Whole exome sequencing in Alopecia Areata identifies rare variants in KRT82

**DOI:** 10.1038/s41467-022-28343-3

**Published:** 2022-02-10

**Authors:** Stephanie O. Erjavec, Sahar Gelfman, Alexa R. Abdelaziz, Eunice Y. Lee, Isha Monga, Anna Alkelai, Iuliana Ionita-Laza, Lynn Petukhova, Angela M. Christiano

**Affiliations:** 1 Department of Genetics & Development, Columbia University, New York, NY USA; 2 Department of Dermatology, Columbia University, New York, NY USA; 3 Institute for Genomic Medicine, Columbia University Irving Medical Center, New York, NY USA; 4 Department of Nutritional and Metabolic Energy, Columbia University, New York, NY USA; 5 Medical Scientist Training Program, Columbia University, New York, NY USA; 6 Department of Biostatistics, Columbia University, New York, NY USA; 7 Department of Epidemiology, Columbia University, New York, NY USA; 8 Present Address: Regeneron Pharmaceuticals, Tarrytown, NY USA; 9 Present Address: MEND Nutrition, Inc., New York, NY USA

**Keywords:** Genomics, DNA sequencing, Autoimmunity

## Abstract

Alopecia areata is a complex genetic disease that results in hair loss due to the autoimmune-mediated attack of the hair follicle. We previously defined a role for both rare and common variants in our earlier GWAS and linkage studies. Here, we identify rare variants contributing to Alopecia Areata using a whole exome sequencing and gene-level burden analyses approach on 849 Alopecia Areata patients compared to 15,640 controls. *KRT82* is identified as an Alopecia Areata risk gene with rare damaging variants in 51 heterozygous Alopecia Areata individuals (6.01%), achieving genome-wide significance (*p* = 2.18E−07). *KRT82* encodes a hair-specific type II keratin that is exclusively expressed in the hair shaft cuticle during anagen phase, and its expression is decreased in Alopecia Areata patient skin and hair follicles. Finally, we find that cases with an identified damaging *KRT82* variant and reduced KRT82 expression have elevated perifollicular CD8 infiltrates. In this work, we utilize whole exome sequencing to successfully identify a significant Alopecia Areata disease-relevant gene, *KRT82*, and reveal a proposed mechanism for rare variant predisposition leading to disrupted hair shaft integrity.

## Abstract (web summary)

Common variants have been discovered to be associated with Alopecia Areata; however, rare variants have been less well studied. Here, the authors use whole-exome sequencing to identify associated rare variants in the hair keratin gene *KRT82*. Further, they find that individuals with Alopecia Areata have reduced expression of KRT82 in the skin and hair follicle.

## Introduction

Alopecia areata (AA) is one of the most prevalent autoimmune diseases, with a lifetime risk of 2.1%, and is characterized by T-cell mediated immune attack of the hair follicle (HF)resulting in hair loss^1^. As a complex disease, both environmental and genetic factors contribute to AA risk^1–3^. Our genome-wide association study (GWAS) and meta-analysis identified common variation in 14 genetic risk loci, supporting the role for common variants in this disease^4,5^. We previously performed linkage analysis in a cohort of families and found statistically significant evidence for rare co-segregating variants^6^, suggesting the presence or rare genetic variants with strong effect sizes. However, the regions we identified were large and precluded the identification of causal genes. Thus, rare genetic variants with strong effect sizes remain uncharacterized in AA.

Whole exome sequencing (WES) and exome-wide association studies in large cohorts of patients with complex disorders such as congenital kidney malformations^7^, amyotrophic lateral sclerosis^8^, pulmonary fibrosis^9^, epilepsy^10^, coronary disease^11^, myocardial infarction^12^, and autism^13^ have successfully identified rare disease variants using WES followed by gene-level collapsing approaches. As variant allele frequencies decrease, the power to detect associations simultaneously decreases due to the lack of observations to inform statistical analyses^14^. As a result, variant-level analysis of WES is limited by the need for large sample sizes to achieve power^15^. Therefore, gene-level collapsing methods have emerged as the accepted framework for identifying genetic burden, the enrichment of variants in a given gene, in disease cases compared to controls^7^. Model parameters, such as variant function and population frequency, are used to select qualifying variants for testing. The frequency of qualifying variants within a gene is compared between cases and controls, and statistical significance is established to determine whether a gene has increased mutational burden in cases.

We performed WES and gene-based collapsing in 849 AA cases and 15,640 controls to assess the genetic burden of rare damaging variants in unknown genes associated with AA.

## Results

### *KRT82* identified as an AA risk gene

**Fig. 1:**
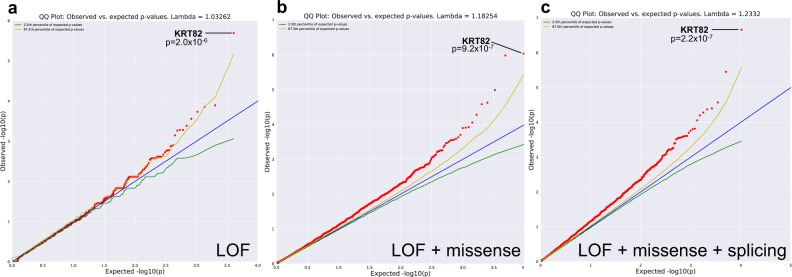
Gene-level collapsing models identify significant rare variation in KRT82. Q–Q plots of **a** Loss of Function (LOF) Model. **b** LOF + damaging missense Model. **c** LOF + missense + splicing variants predicted by high TraP scores (≥0.2). The genomic inflation factor (λ) is 1.03, 1.18, 1.23, respectively. The blue line represents the null hypothesis (observed *p*-values correspond to expected *p*-values), the yellow line marks the 2.5th percentile of expected p-values, and the green line marks the 97.5th percentile of expected *p*-values. *p*-value was determined by two-tailed Fisher’s exact test (FET).

In the first collapsing model, we performed a gene-based analysis by evaluating only rare, loss of function (LOF) variants with a minor allele frequency (MAF) ≤ 1% in the general population (gnomAD populations) and our sequencing cohorts (cases and internal controls)^16^. Stringent quality control (QC) metrics were further imposed on the variants (see “Methods”). Using this approach across 18,653 protein-coding genes, we identified Keratin 82 (*KRT82)* as the only AA case-enriched gene at genome-wide significance (at a significance level 0.05/18,653 = 2.68 × 10E−06) (OR = 4.04, *p* = 2.03 × 10E−06). We detected variants in 19/849 AA cases (2.24%), compared to 88/15,640 controls (0.56%) (Fig. 1a, Table 1). *KRT82* is predicted to be tolerant to protein truncating variants due to the increased frequency of observed LOF variants compared to the number expected by chance in the general population (gnomAD, pLI = 0; o/e = 1.12)^16^. Due to the predicted tolerant nature of *KRT82*, we investigated whether the stringent frequency threshold for rare variants in the LOF model excluded common variants that would have altered the *KRT82* burden in cases and controls. To address this, we performed an additional analysis on common LOF variants (MAF > 1%) to ensure that the finding of an increased frequency of LOF *KRT82* variants in cases was not an artifact of excluding common variants found in either cases or controls. Our supplementary model tested whether *KRT82* variants were truly enriched in AA cases compared to controls, and not a function of selecting for only rare variants and disregarding other variation that may be present at the locus. In confirmation of a true association between *KRT82* variation and AA patients, the common (MAF > 1%) LOF analysis did not identify additional variants in *KRT82*. This indicated that the rare model accounted for all relevant LOF variants at the *KRT82* locus and that AA patients harbored an enriched frequency of *KRT82* variants compared to controls. The rare LOF model also identified another gene that encoded a hair keratin-related protein, *KRTCAP3* (Keratinocyte Associated Protein 3) as a second AA association (*p* = 1.28E−04) with rare LOF qualifying variants in 9/849 (1.1%) AA cases and 30/15,640 (0.19%) controls (Table 1). *DECR2*, which encodes an enzyme of beta-oxidation, was the third most significantly identified gene in the LoF model (p = 1.39E−04), however, testing in a larger cohort may be needed to achieve a genome-wide significant association of these genes with AA.

**Table 1:** Case and control frequencies of collapsing models’ qualifying variants in the top three most significant genes.

Model	Gene	Cases	Controls	Case frequency	Control frequency	*P*-value
LoF	KRT82	19	88	2.2%	0.6%	2.03E−06
	KRTCAP3	9	30	1.1%	0.2%	1.28E−04
	DECR2	6	11	0.7%	0.01%	1.39E−04
LoF + missense	KRT82	47	376	5.5%	2.4%	9.19E−07
	ZNF418	0	271	0%	1.7%	1.04E−06
	PCCB	2	321	0.2%	2.1%	1.02E−05
LoF + missense + splicing	KRT82	51	404	6.0%	2.6%	2.18E−07
	NCOA3	4	419	0.4%	2.7%	3.54E−06
	IGFBL1	0	214	0%	1.4%	2.67E−05

*p*-value was determined by two-tailed Fisher’s exact test (FET).

Next, we used a second gene-based collapsing model that expanded upon the previous model to include rare variants (MAF ≤ 1%) that were non-synonymous coding (missense) or canonical splice variants, and were predicted to be damaging by PolyPhen‐2 HumVar^17^. The inclusion of damaging missense variants strengthened the evidence for *KRT82* association (*p* = 9.19 × 10E−07, Fig. 1b, Table 1), and identified qualifying variants in 47/849 cases (5.54%) versus 376/15,640 controls (2.4%; OR = 2.38). This indicated that the *KRT82* association with AA was driven by both protein truncating variants (using the LOF model), as well as by missense variants that altered the protein structure (using the LOF + missense model).

We postulated that if *KRT82* was enriched for both LOF and damaging missense variants, a stronger signal would be observed with the inclusion of qualifying synonymous and splice region variants predicted to be pathogenic. We used the Transcript-inferred Pathogenicity score (TraP-score, version 2.0)^18^, which predicts whether a synonymous or intronic variant can damage the mRNA transcript by disrupting the splicing process, resulting in perturbed protein formation and subsequent disease risk^19–21^. We constructed an additional gene-collapsing model (Fig. 1c, Table 1) that expanded on the previous LOF and missense models to include predicted pathogenic splice variants and non-synonymous variants with a TraP-score ≥ 0.2, which is above the 90th percentile of pathogenic scores and considered possibly damaging (http://trap-score.org/about.jsp). Notably, this model also identified *KRT82* as the gene harboring the most significantly enriched variants in AA cases, achieving a stronger enrichment of *p* = 2.2 × 10E−07, OR = 2.41 (Fig. 1c, Table 1) and surpassing genome-wide and study-wide thresholds for significance (α = 2.68E−06 and 3.83E−07 respectively; see “Methods”). The increased significance was due to an additional splice region variant (Arg314Gln) that had a high TraP-score (0.55) prediction as protein-damaging and was found in four additional cases. As a result, this splicing model identified a total of 51/849 AA patients (6.01%) with rare damaging variants in *KRT82*, compared to 404/15,640 controls (2.58%).

We found that the genomic inflation factor (λ), which measures potential false-positive associations, varied across these models in a manner that was consistent with the variant type included in each model. For example, LOF variants have a higher probability of exerting a phenotypic effect than other variant types^22,23^. Accordingly, we observed the lowest estimate of genomic inflation for the LOF model (λ = 1.03) (Fig. 1a). We found greater estimates for the LOF + missense analysis (λ = 1.18) and the splicing analysis (λ = 1.23), models for which we expected a higher proportion of clinically benign qualified variants (Fig. 1c). To investigate further, we tested an additional model as a negative control, using rare synonymous variants as qualifying criteria (MAF ≤ 0.01%). As expected, we did not observe any genes with significant enrichment. The genomic inflation factor (λ) for this model was 0.91 (Supplementary Fig. 1, Supplementary Table 1). Taken together, the overall low inflation of the cohort in the synonymous analysis represented the null distribution and absence of global inflation due to genotyping discrepancies or population stratification. It provided further confidence for a true association between rare, damaging *KRT82* variants and AA.

Qualifying variants in the combined gene-collapsing models included predicted damaging variants such as stop-gained (nonsense), frameshift, splice site, and nonsynonymous damaging amino acid substitutions (missense) (Table 2). Eleven protein-altering variants (3 nonsense, 7 damaging missense, 1 splicing) accounted for the variation detected in 51 of our AA cases (Table 2). One *KRT82* variant with the highest frequency in our AA cohort was found in 15 cases (1.77%) and resulted in a nonsense mutation at amino acid position 47 (R47X). Furthermore, 7 out of the 11 variants were shared among more than one individual. The pathogenicity of these individual variants was supported by their recurrent nature and increased frequency in our disease cohort, even though the variants were rare (MAF ≤ 1%) in the general population. However, none of the individual variants were capable of exceeding the genome-wide significance threshold on their own, underscoring the necessity of gene-level analysis in obtaining sufficient power to identify meaningful disease risk genes.

**Table 2:** Eleven damaging amino acid changes in *KRT82*, the case and control frequencies and *p*-values.

Type	Cases	Controls	Case frequency	Control frequency	*P*-value
**LoF**	**19**	**88**	**2.24%**	**0.56%**	**2.03E**−**06**
Arg47X	15	72	1.77%	0.46%	3.42E−05
Ser27X	3	3	0.35%	0.02%	2.42E−03
Ser16fs	1	3	0.12%	0.02%	0.19
**LoF** + **missense**	**47**	**376**	**5.54%**	**2.40%**	**9.19E**−**07**
Asn129Lys	14	101	1.65%	0.65%	2.34E−03
Ile92Val	4	46	0.47%	0.29%	0.33
Glu351Lys	4	37	0.47%	0.24%	0.16
Arg314Trp	3	22	0.35%	0.14%	0.14
Arg302His	1	7	0.12%	0.04%	0.34
Gly436Arg	1	2	0.12%	0.01%	0.15
Gly436Trp	1	2	0.12%	0.01%	0.15
**LoF** + **missense** + **splicing**	**51**	**404**	**6.01%**	**2.58%**	**2.18E**−**07**
Arg314Gln	4	28	0.47%	0.18%	0.08

List of the 11 qualifying variants present in AA cases, sub divided by the collapsing model that identified the corresponding qualifying variants and subsequent amino acid changes (bolded). Each model includes qualifying variants from the previous model. For example, LOF + missense model includes the 3 LOF variants in 19 cases, plus 7 missense variants in 28 new cases, for a total of 10 variants in 47 cases. Qualifying variants exclusive to controls (*n* = 50) were not listed in this table but accounted for the total number of controls with a qualifying variant (Controls column). *p*-value was determined by two-tailed Fisher’s exact test (FET).

### AA *KRT82* variants annotated as likely disease-causing

The identification of *KRT82* as an AA risk gene emphasized the utility of WES in uncovering functionally relevant disease loci in complex diseases. *KRT82* encodes a type II hair-specific keratin with exclusive expression in the cuticle of the hair shaft^24–26^. Keratins are intermediate filaments (IF) that provide a cellular structural network via the heterodimerization of type I with type II keratins in epithelial cells^27^. The keratin family of proteins are composed of well-defined evolutionarily conserved domains consisting of a head domain, a 310-amino acid α-helical rod domain, and a tail domain^28^. The rod domain is necessary for forming the coiled-coil α-helical dimer between type I and II keratins, and is made up of 4 subdomains, 1A, 1B, 2A, 2B^28,29^. Additionally, the keratin II head domain is crucial for tetramer stabilization and IF assembly^30^. Variants that disrupt the 1A, 2B, or type II head and tail domains in keratins have been reported as pathogenic in various other diseases, underscoring the importance of these domains in proper keratin function^28,31^.

**Fig. 2:**
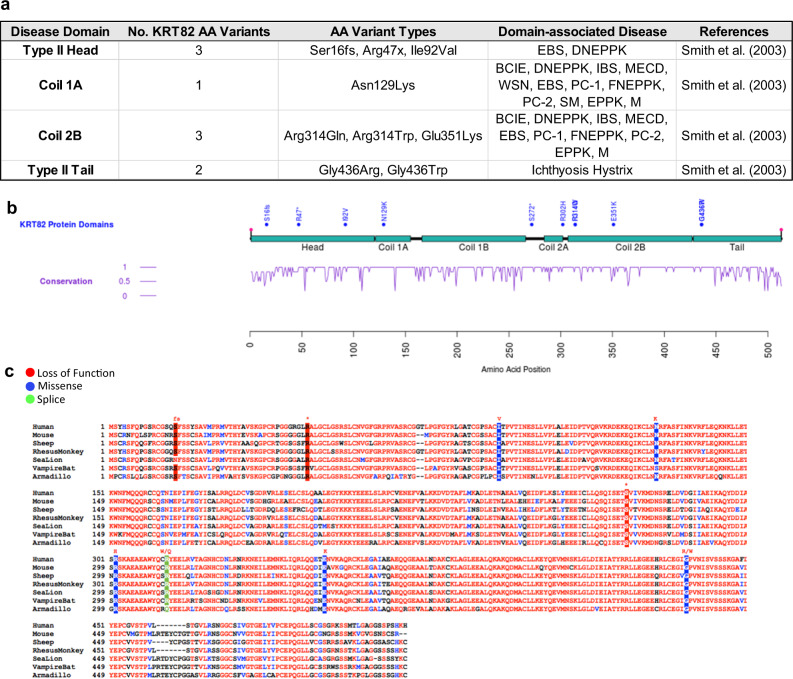
KRT82 variants are localized to highly conserved disease-annotated domains. **a** Table of 9 of the KRT82 damaging variants that fall in a domain previously associated with pathogenic keratin variants in other diseases. **b** schematic representation of *KRT82* with domains (turquoise), AA variants (blue), and conservation score (purple) (1 = highly conserved). **c** Conservation of *KRT82* amino acids across 7 different species: human, mouse, sheep, rhesus monkey, sea lion, vampire bat, and armadillo. AA variants are highlighted in red (LoF), blue (Missense), and green (Splicing). Amino acid change of variant is annotated in red above. EBS = epidermolysis bullosa simplex; CC = Cryptogenic Cirrhosis; DNEPPK = diffuse non-epidermolytic palmoplantar keratoderma; BCIE = bullous congenital ichthyosiform erythroderma; IBS = ichthyosis bullosa of Siemens; MECD = Meesmann’s epithelial corneal dystrophy; WSN = white sponge nevus; PC = pachyonychia congenita; FNEPPK = focal non-epidermolytic palmoplantar keratoderma; SM = steatocystoma multiplex; EPPK = epidermolytic palmoplantar keratoderma; M = monilethrix.

We analyzed the location of 11 rare, damaging variants in the *KRT82* gene that contributed to the association with AA (Table 2). Strikingly, 9 out of the 11 *KRT82* variants fell within one of the keratin disease-associated subdomains, consistent with the predicted damaging effect of these variants (Fig. 2a, b). Three *KRT82* variants resided in the head domain (Ser16fs, Arg47X, Ile92Val), one in coil 1A (Asn129Lys), three in coil 2B (Arg314Gln, Arg314Trp, Glu351Lys), and two in the tail domain (Gly436Arg, Gly436Trp) of *KRT82* (Fig. 2a, b). Since these regions are essential for keratin function and dimerization, we postulated that the *KRT82* AA risk variants likely disrupt KRT82 function and dimerization with its respective potential binding partners (KRT32, KRT35, KRT39, or KRT40). Therefore, missense and LOF variants in these well-defined disease-associated regions have the potential to impair normal IF assembly and disrupt the integrity of keratins in the hair shaft cuticle in AA cases.

Additionally, all identified damaging variants fell within evolutionarily conserved residues across distantly related species, further supporting the importance of these amino acids in KRT82 biology (Fig. 2c). Highly conserved genes are often considered intolerant to variation and undergo negative purifying selection against deleterious alleles^32^. Since important functional residues are often evolutionarily conserved across species, variants occurring at these positions are more likely to be pathogenic^32–34^. The enrichment of *KRT82* variants in established functional disease domains and evolutionarily conserved amino acids supported a deleterious effect of these variants on KRT82 function.

### *KRT82* variants associated with AA pathology

Within the large family of keratins, the hair follicle is composed of subfamilies of keratins (type I and type II) exclusively expressed in the hair follicle; the “soft” epithelial keratins (*KRT25*-*28*, *KRT71*-*75*) and the “hard” keratins (*KRT31*-*40*, *KRT81*-*86*) responsible for the keratinization of hair and nails^24,27^. Interestingly, mutations in type II hard and soft hair keratins (*KRT75*, *KRT81,83,85,86*), but not type I hair keratins (*KRT25-28, KRT31-40)*, have reported associations with other genetic hair disorders such as Monilethrix, pseudofolliculitis barbae, and hair ectodermal dysplasia^26,31,35^. Specifically, mutations in the 1A and 2B domains of *KRT81, KRT83*, and *KRT86* resulted in Monilethrix, a rare monogenic hair loss disorder (Fig. 2a)^28,35^. The causal role of type II hair keratins in genetic hair diseases validated their important role in hair structure and further strengthened the functional association between AA and *KRT82*, a type II hair keratin.

**Fig. 3:**
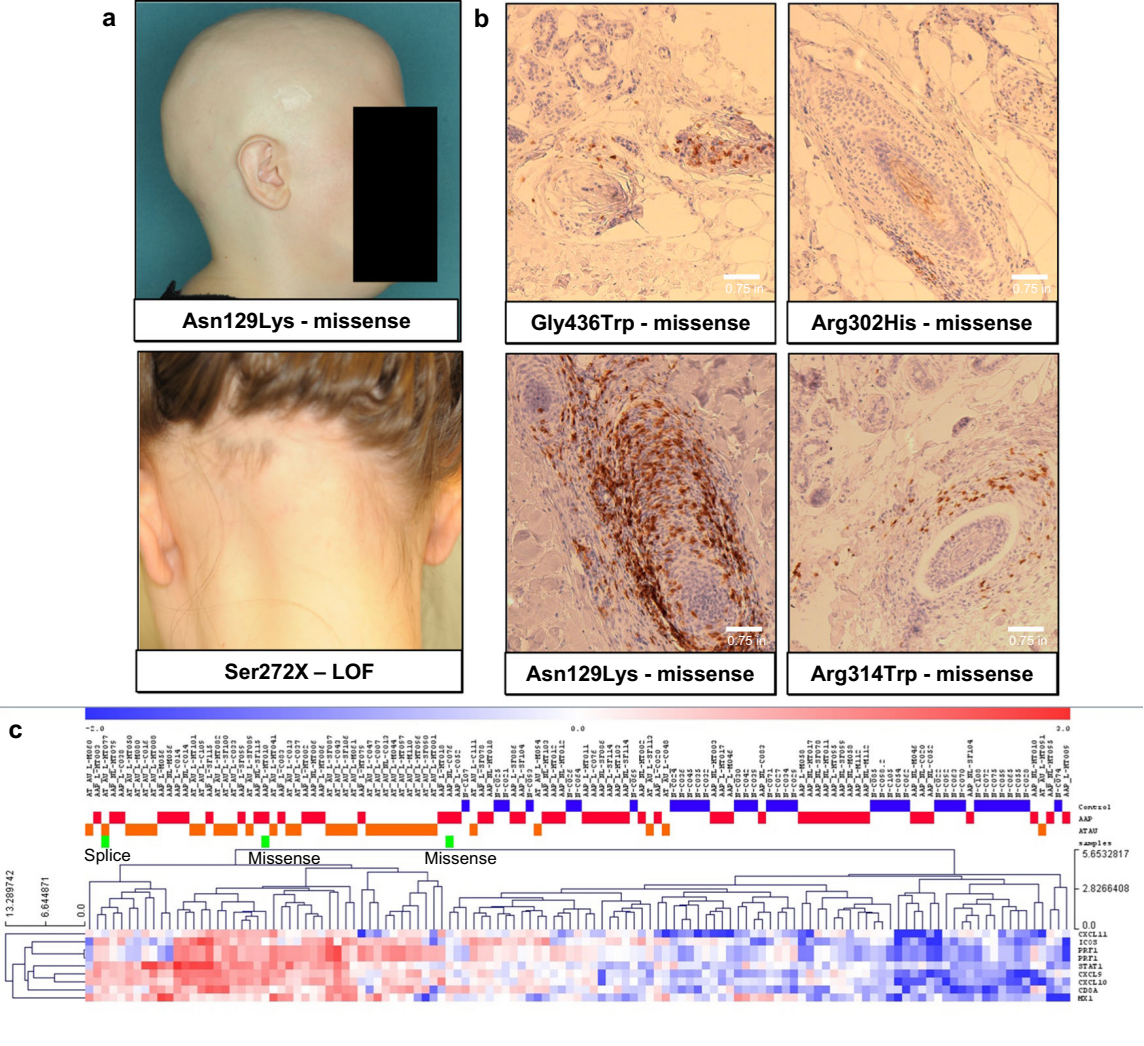
Patients with KRT82 variants confirmed to have AA. **a** Clinical photos of two patients with a missense and nonsense (LOF) variant in KRT82, respectively. Both show hair loss patterns characteristic of AA. **b** Immunohistochemistry (IHC) staining of CD8+ T cells in 4 patients with missense variants in *KRT82*. Perifollicular CD8+ T cell infiltrate is a diagnostic characteristic of AA. Each biopsy shown is taken from an individual with AA, serving as 4 biological replicates. **c** RNA expression profiles of the ALADIN (Alopecia Areata Disease Severity Index) inflammatory signature confirms that three patients (green bars) with KRT82 variants (splice and missense) have increased expression of the inflammatory signature, characteristic of AA. Additionally, these three KRT82-carrying patients (green) cluster within AA patients (AAP-red, ATAU-orange), and not with controls (blue).

Due to the known roles of other type II hair keratin genes in other monogenic hair loss disorders, we next confirmed that our patients were accurately diagnosed with AA, and not a monogenic hair loss disorder misdiagnosed as AA in our cohort. We confirmed accurate AA diagnoses through clinical validation, biopsy immunohistochemistry (IHC), and inflammatory ALADIN gene expression signatures (Fig. 3a–c). In four AA patients with a *KRT82* missense variant, IHC of scalp samples revealed interfollicular infiltrate of CD8+ lymphocytes, a diagnostic feature of AA (Fig. 3b). The ALADIN (Alopecia Areata Disease Severity Index) index utilizes expression profiles of keratin and inflammatory gene signatures to distinguish AA scalp from control^36^. Gene expression data from three patients with a *KRT82* variant confirmed an increased inflammatory ALADIN signature, with the patients clustering in the AA cohort and away from controls (Fig. 3c). Using these three methods, we confirmed that diagnosis of AA was accurate, and did not represent a form of a monogenic hair loss disorder.

### AA susceptibility is specific to *KRT82* variation

**Fig. 4:**
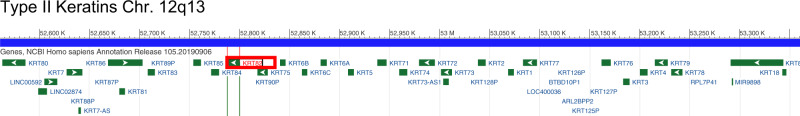
Type II keratin genes cluster together on Ch12. 700 kb chromosomal locus encompassing the type II keratin gene cluster (green bars) on chromosome 12. *KRT82* is boxed, in red.

Due to the close co-localization and structural similarities within the keratin gene family, we tested whether the increased variation in *KRT82* was due to hypermutability in the chromosomal region or the keratin family of genes. We applied the same model parameters to specifically investigate variant frequencies in 7 other keratin genes (*KRT5, KRT84, KRT85*, *KRT32, KRT35, KRT39, KRT40*) located both proximally and distally to *KRT82* (Fig. 4, Table 3). *KRT84* and *85* are type II hair keratins located 8.3 kbp and 26.4 kbp upstream of *KRT82*, respectively. *KRT5* is a type II epithelial keratin gene located on chromosome 12, 120.6 kbp away^37^. *KRT32, KRT35, KRT39, KRT40* are all type I hair keratins and proposed binding partners of *KRT82* due to their reported co-expression patterns in the HF cuticle^27^. Each of these keratin genes harbored markedly less variation than *KRT82*. For example, only one AA patient carried a LOF variant (*KRT85*) out of the seven additional keratin genes queried, whereas 19 AA cases harbored a *KRT82* LOF variant (Table 3). Furthermore, none of the additional keratin genes we investigated carried a significantly higher genetic burden of rare variants in cases compared to controls in any of the collapsing models. The lack of significant AA-associated variation in any of the putative binding partners of *KRT82* or family members suggested a specific role for *KRT82* in the genetic pathomechanism in AA. Additionally, these results confirmed that the AA-associated risk is driven specifically by *KRT82* variation, and was not a result of broad hypermutability across the keratin gene family or chromosome 12 block.

**Table 3:** Significant AA-associated variation is specific to *KRT82* in comparison to seven other keratin genes including four type I keratins (*KRT32, KRT35, KRT39, KRT40*), two type II hair keratins (*KRT84, KRT85*), and one type II epithelial keratin (*KRT5*).

Type	Cases	Controls	Case frequency	Control frequency	*P*-value
LoF					
*KRT82*	19	88	2.24%	0.56%	2.03E−06
*KRT84*	0	13	0.00%	0.08%	1
*KRT85*	1	3	0.12%	0.02%	0.1906
*KRT5*	0	1	0.00%	0.01%	1
*KRT32*	0	4	0.00%	0.03%	1
*KRT35*	0	3	0.00%	0.02%	1
*KRT39*	0	16	0.00%	0.10%	1
*KRT40*	0	29	0.00%	0.19%	0.4014
LoF + missense					
*KRT82*	47	376	5.54%	2.40%	9.19E−07
*KRT84*	25	428	2.94%	2.74%	0.6668
*KRT85*	8	159	0.94%	1.02%	1
*KRT5*	3	80	0.35%	0.51%	0.8012
*KRT32*	6	111	0.71%	0.71%	1
*KRT35*	12	269	1.41%	1.72%	0.5869
*KRT39*	8	127	0.94%	0.81%	0.693
*KRT40*	17	267	2.00%	1.71%	0.4973
LoF + missense + splicing					
*KRT82*	51	404	6.01%	2.58%	2.18E−07
*KRT84*	25	457	2.94%	2.92%	0.9168
*KRT85*	25	344	2.94%	2.20%	0.999
*KRT5*	6	144	0.71%	0.92%	0.709
*KRT32*	6	142	0.71%	0.91%	0.7081
*KRT35*	12	284	1.41%	1.82%	0.5053
*KRT39*	8	127	0.94%	0.81%	0.693
*KRT40*	18	267	2.12%	1.71%	0.3438

Values denote the number of rare damaging variants in seven other keratin genes: four type I keratins (*KRT32, KRT35, KRT39, KRT40*), two type II hair keratins (*KRT84, KRT85*), and one type II epithelial keratin (*KRT5*). None of the other keratins reach significance in any of the 3 models. Type II keratins were chosen for their similar classification and positional proximity to *KRT82*. The type I hair keratins are the putative binding partners of *KRT82*. *p*-value was determined by two-tailed Fisher’s exact test (FET).

### Haplotype analysis identified hotspot mutation R47X in *KRT82*

**Fig. 5:**
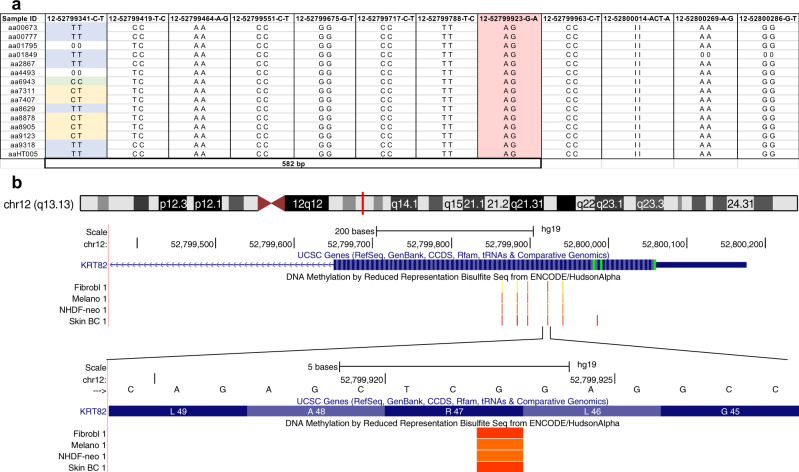
Haplotype analysis identifies Arg47X as a hotspot mutation. **a** 15 AA patients heterozygous for the Arg47X variant (12-52799923-G-A; highlighted in pink). Different genotypes (blue = alternate homozygous, yellow = heterozygous, green = reference homozygous) at variant 12-52799341-C-T (582 bp away) confirms the presence of the Arg47X A allele on different haplotypes, identifying it as a hotspot mutation. **b** Schematic of *KRT82* exon 1 (blue) with ENCODE methylation patterns (orange) in 4 skin cell types: fibroblasts (Fibrobl), Melanocytes (Melano), Neonatal Dermal Fibroblasts (NHDF-neo), and Skin Tissue (Skin BC). This is a visualization of the methylated cytosine (C) of the sense strand that is spontaneously deaminated resulting in a mutational hotspot and premature stop codon at position 47.

We next asked whether the *KRT82* variant R47X is a hypermutable hotspot versus a founder variant that was dispersed across the population. Some regions of the genome have a propensity to spontaneously give rise to de novo variants, increasing the frequency of disease variants in the population. For example, another dermatologic disorder, epidermolysis bullosa simplex (EBS), is associated with hotspot mutations in keratin genes *KRT5* and *KRT14* that result in defective cross-linking and bundle formation^38–40^. For a hotspot mutation, the same variant will be carried on multiple different haplotypes, whereas for a founder variant, it would be found on one common haplotype, suggesting the presence of a common ancestor. To distinguish these possibilities, we analytically phased our sequence data to identify haplotypes carrying *KRT82* variants^41^. We found that the most frequently occurring nonsense variant, R47X was present on at least six different haplotypes, indicative of a hotspot mutation (Fig. 5a, Supplementary Table 2).

Disease-associated mutational hotspots commonly occur at CpG dinucleotides that result in spontaneous deamination of 5-methylcytosine in C > T transitions^42–46^. Interestingly, the R47X variant in KRT82 resulted from a C > T transition at a methylated CpG residue (Fig. 5b). In this context the 5′-CGA-3′ encoded an arginine (Arg; R) that resulted in a nonsense variant when 5′-methylcytosine was deaminated to thymine (T). As a result, R47X, the most frequent KRT82 variant in AA patients (15/849; 1.77%), is a hotspot mutation arising from a spontaneous deamination at a methylated CpG site.

### KRT82 is exclusively expressed in anagen phase

**Fig. 6:**
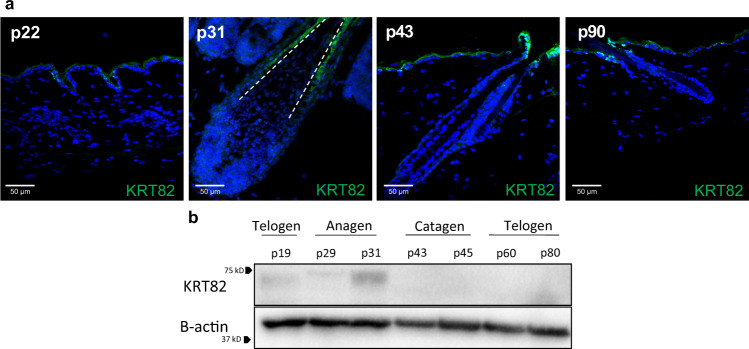
KRT82 is expressed exclusively in anagen. **a** IF of KRT82 (green) in postnatal (p) mouse skin. Mouse HFs are in telogen at postnatal days 22 and 90 (p22, p90), in anagen at p31, and catagen at p43. Cuticular staining of KRT82 is only observed in the p31 (anagen) HF. White dashed line outlines the hair shaft cuticle. Weak staining observed in mouse epidermis may be a result of green autofluorescent properties of skin epidermal cells. IF staining was repeated at 3 other anagen timepoints and 2 additional telogen timepoints with similar results. **b** Western blot analysis of whole mouse skin at varying stages of the hair cycle shows that keratin protein is only present in the anagen phase (p31) of the mouse hair cycle. Western blot was repeated with similar results 2 additional times. Source data are provided as a Source data file.

Previous studies reported the expression of KRT82 in the hair shaft cuticle of both human and sheep hair follicles, and identified it as a hair-specific type II keratin^24–26^. However, its functional role in hair follicle biology has yet to be elucidated. Cell culture studies investigating the role of KRT82 are challenging due to its differentiated nature as a hard hair follicle keratin, therefore, we focused functional analyses on in vivo mouse models. We characterized the spatiotemporal expression of KRT82 throughout the murine hair follicle cycle, which consists of the three stages including anagen (growth), catagen (regression), and telogen (quiescence). We found that KRT82 was expressed in the hair follicle cuticle exclusively during anagen (Fig. 6), and was absent in catagen and telogen. Thus, KRT82 expression in anagen is biologically relevant in the context of AA, since the autoimmune attack is known to occur exclusively during anagen, resulting in the premature entry of the HF into catagen.

### Reduced expression of KRT82 in AA patients

**Fig. 7:**
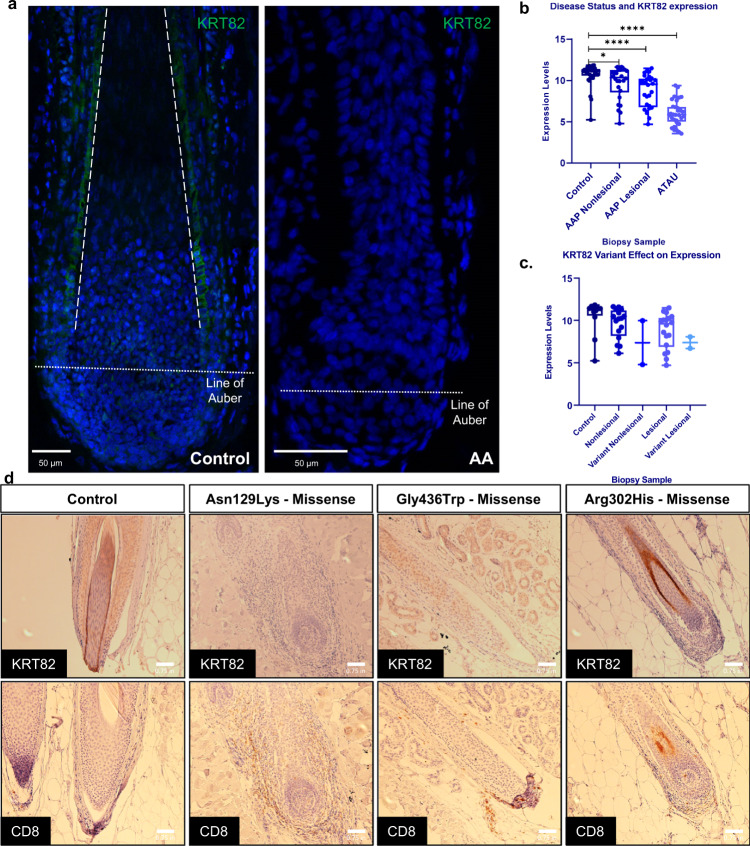
Functional KRT82 expression is reduced in AA HF and skin. **a** IF staining of KRT82 (green) in human healthy control and AA HF. Dashed line outlines the cuticle, dotted line represents the Line of Auber. IF staining with similar results was repeated in 1 additional control sample and 2 additional AA samples (biological replicates) **b** RNA expression of KRT82 from whole skin scalp biopsies of control, AAP nonlesional sites, AAP lesional sites, and AT/AU sites. Each circle represents one sample. Statistical analysis was performed using two-tailed unpaired *t*-test with Welch’s correction. *p = 0.028, ****p < 0.0001. *n* = 98 individuals (36 controls, 30 AAP, 32 ATAU). **c** Scalp RNA expression of patients carrying *KRT82* variants at nonlesional (Variant Nonlesional) and lesional (Variant Lesional) sites compared to AA patients without *KRT82* variants (Nonlesional and Lesional) and controls without AA or *KRT82* variants. *n* = 32 individuals (12 controls and 20 AAP patients). Expression data is displayed in boxplots in which each sample data point is represented by individual dots; the center line denotes the median; upper and lower limits of the box denote 75th and 25th percentiles, respectively; and the whiskers indicate the dataset maximum and minimum. **d** IHC of KRT82 and CD8 in healthy control and lesional biopsies in three AA patients with missense variants. AA biopsies are from individual patients and serve as 3 biological replicates. Staining in another control biopsy was not repeated; however, images were taken of other hair follicles, serving as technical replicates.

Since type I and type II hair keratins are responsible for the “hard” keratinization of hair and nails^24,27^, we next investigated the expression of KRT82 in control human hair follicles using immunofluorescence (IF) staining and found strong expression of KRT82 in the anagen hair shaft cuticle (Fig. 7a), as previously reported. Prior work investigating the expression of hair keratins in the nail unit found that KRT82 was not expressed in any components of the nail in health adults^47^. In control HFs, KRT82 cuticle expression began in the bulb just above the Line of Auber, and extended up the HF toward the epidermis. We found specific subcellular KRT82 localization in the cytoplasm of the hair shaft cuticle cells, consistent with the role of keratins as cytoskeletal intermediate filaments (IF)^28^. We compared KRT82 expression in control versus AA patient HFs and found markedly decreased expression of KRT82 in AA hair shaft cuticles (Fig. 7a). In contrast to control HF, KRT82 expression in AA follicles was absent in the cuticle including the bulb, the site of AA immune attack. This finding is noteworthy in the context of AA disease onset due to the characteristic immune infiltrate surrounding the follicular bulb, where we identified loss of functional KRT82 expression in AA HF.

In support of this observation, our previous gene expression studies reported a significant −4.5 log-fold decrease in expression of KRT82 in the skin of AA patients compared to controls (Benjamini–Hochberg-corrected *p* = 3.55E−10)^36^. We found that not only was KRT82 expression significantly decreased in AA patients relative to controls, but KRT82 downregulation was also significantly correlated with disease severity (Fig. 7b). Biopsies taken from nonlesional (hair-bearing) sites of AA Patchy (AAP) scalp skin revealed a slight reduction in KRT82 expression compared to controls (*p* = 0.028) (Fig. 7b). In lesional sites of hair loss in AAP patients and patients with total loss of scalp and body hair (AT/AU; alopecia areata totalis/universalis), KRT82 was significantly decreased in patient scalp biopsies compared to healthy controls (*p* < 0.0001) (Fig. 7b). Due to the fact that KRT82 is expressed exclusively in the hair follicle, we investigated whether the observed decrease in expression was simply a consequence of loss of the hair follicle, the organ expressing KRT82. However, the insignificant change in KRT82 levels in AAP nonlesional compared to lesional biopsy sites (*p* = 0.068) suggested that decreased expression observed in patients was not a result of the absence of HFs, but rather a predisposed reduction in functional KRT82 expression that strongly correlated with active disease sites and disease severity (Fig. 7b). However, it cannot be ruled out that more significant differences between nonlesional and lesional samples may be observed with a larger sample size. Our results suggested that decreased functional KRT82 in the hair shaft cuticle may predispose genetically susceptible individuals to AA-mediated hair loss.

To investigate whether the rare, damaging variants effected expression of *KRT82* in the skin, we tested whether individuals with qualifying *KRT82* variants demonstrated altered KRT82 levels in scalp skin in 20 AAP individuals. Due to the rare frequency of the *KRT82* variants, only 2 of the 20 patients harbored a damaging *KRT82* variant, for which we obtained both lesional and nonlesional biopsies. We found that the patients with a confirmed damaging variant in *KRT82* trended towards decreased expression of KRT82, independent of biopsy type (Fig. 7c); however, the difference was not statistically significant due to the small sample size. The trending decreased expression in individuals with a *KRT82* variant suggested that the identified damaging variants had a functional effect on scalp expression in AA patients by potentially altering or truncating the KRT82 protein structure, resulting in decreased functional expression in AA skin. These results should be interpreted with caution due to the small number of *KRT82* variant-bearing patients with scalp skin expression data.

We next characterized the functional effect of *KRT82* variants in the HF by assessing KRT82 and CD8 expression in lesional scalp biopsies of three patients carrying one of the damaging *KRT82* variants. Lesional biopsies from two of the patients with a missense *KRT82* variant (Asn129Lys, Gly436Trp) revealed reduced or absent expression of heterozygous KRT82 in the HF cuticle (Fig. 7d). Both patients also had increased CD8+ T cell follicular infiltrate compared to control HFs. In the third patient with a missense *KRT82* variant (Arg302His), we observed HFs with cuticular KRT82 expression, however, with less prominent perifollicular CD8+ T cell infiltrate (Fig. 7d) compared to the patients with absent KRT82. These findings suggest a model in which rare damaging variants in *KRT82* resulted in reduced protein function or expression, predisposing AA patients to compromised HF cuticle structure, and rendering the HF vulnerable to autoimmune attack. This is supported by the observed negative correlation between perifollicular CD8+ T cell infiltrate and KRT82 HF cuticle expression.

## Discussion

While whole genome sequencing (WGS) provides greater coverage and sensitivity, whole exome sequencing (WES) is a more cost-effective alternative that has been successful in identifying rare variants in complex diseases. Previous studies in amyotrophic lateral sclerosis (ALS), myocardial infarction, Alzheimer’s disease, and schizophrenia have been successful in identifying significant contributions of rare variants using WES^8,12,48,49^. This study presents the first large-scale WES of AA patients and identification of previously unknown rare variants that contribute to disease risk. We used WES and gene-level collapsing analyses to identify a type II hair keratin gene, *KRT82*, as the only gene harboring significantly more rare damaging variants in AA cases (6.01%) compared to controls (2.58%) (*p* = 2.18E−07), achieving both genome-wide and study-wide significance. In our previous GWAS and meta-analysis, we did not detect significant association in the *KRT82* region likely due to the inability to detect rare variants using GWAS. The predicted damaging effects of the WES- identified rare *KRT82* variants were supported by their localization to evolutionarily conserved residues and known disease-causing keratin domains.

In order to define the underlying mechanism for the association between *KRT82* and AA, we interrogated the role of KRT82 in hair follicle biology. We observed that KRT82 is expressed in the hair shaft cuticle exclusively in anagen, when the AA-directed immune attack occurs. The absence of KRT82 in AA hair shaft cuticles may cause HF dysfunction, immune infiltration, and subsequent premature entry into catagen. In support of this notion, hair follicles in another model, *KRT17*-null mice, exhibit hair shaft fragility, aberrant apoptosis, and premature entry into catagen^50^. In the absence of *KRT17*, keratinocytes are more susceptible to TNFα-mediated apoptosis and HFs prematurely undergo the anagen-catagen transition, demonstrating the critical role for a single keratin, *KRT17*. Further support for the role of *KRT82* in hair loss is seen in the *Sox21* knockout (*Sox21*^*−/−*^*)* mouse model of cyclic alopecia that demonstrated altered keratin expression signatures, including a prominent reduction of KTR82 in the hair shaft cuticle^25^. The resulting phenotype led to hair loss during late first telogen and catagen stages. Although other keratin levels were affected in this animal model, the decrease in KRT82 and association with cyclical alopecia substantiates an important role for *KRT82* in hair follicle biology, and underscores the impact of LOF and damaging variants in AA pathogenesis.

Further functional annotation of *KRT82* revealed that its expression was largely reduced in the scalp skin biopsies and HFs of AA patients. All 51 AA patients in which variants were detected are heterozygous carriers of damaging risk variants, which correlated with lower levels of KRT82 expression supporting a haploinsufficiency model. The absence or reduced expression of KRT82 in the HF, in addition to increased perifollicular CD8+ T cells support an important relationship between KRT82 function and the organ-specific AA-mediated immune attack. We postulate that hair shaft cuticle formation is dependent on KRT82 and absence of normal expression renders the bulb vulnerable to infiltration of auto-reactive immune cells.

As the outermost layer of the hair shaft, the hair shaft cuticle protects the internal layers of the HF against mechanical and environmental insults through a high content of crosslinking between type I and type II keratins^51^. It also plays a crucial role in anchoring the growing hair shaft to the inner root sheath (IRS) during anagen^51^. Notably, disrupted and absent hair shaft cuticles are observed in exclamation mark hairs (EMH), a pathognomonic diagnostic feature of AA^52^. Previous reports demonstrated that absent cuticles in AA EMHs revealed areas of increased levels of hair shaft disorganization^52^. Additionally, some hairs revealed structural cuticle deformities with a high density of vacuoles^52^. The authors suggested that the cuticle disruption negatively impacts AA HF integrity, causing vulnerability to mechanical insults^52^. Our finding of a genetic association of damaging *KRT82* variants with AA, and the presence of aberrant hair shaft cuticles in AA EMHs, supports a critical role for the hair shaft cuticle in AA pathogenesis.

Although the target antigens in AA have not been conclusively identified, keratins are attractive candidate auto-antigens due to their abundant expression in the HF. Auto-antibodies against anagen cuticle, matrix, and cortex keratinocytes were previously identified in a subset of autoimmune polyendocrine syndrome type 1 patients with alopecia areata totalis (AT)^53^. Human and mouse AA auto-antibody reactivity was reported against anagen HF-specific antigens ranging from 40–60 kDa, specifically 44/46 kDa keratins^53–55^. Type I keratins are 44–48 kDa and dimerize with type II keratins (55–60 kDa) to form IF bundles and structural network in cells^24,27^. In AA, absence of the type II keratin, KRT82 in the cuticle would leave its type I binding partner (KRT32, KRT35, KRT39, or KRT40) unable to heterodimerize. We postulate that without functional KRT82, unbound type I binding partners have the potential to be aberrantly presented as auto-antigens, and invites further work on this question.

Here, we used WES and gene-level variant analyses to identify a functionally relevant disease gene, *KRT82*. We identified eleven rare damaging variants, including a frequent mutational hotspot (R47X), and characterized the negative consequences on KRT82 expression and function in AA. Our work proposes a mechanism involving genetic susceptibility to a disrupted hair shaft cuticle structure, and subsequent susceptibility to AA. This work not only elucidates a previously unknown mechanism of end organ genetic susceptibility in an autoimmune disease, but also invites potential new therapeutic approaches for treatment of AA. Treatments targeted at restoring HF integrity may be advantageous due to the distinct ability of the HF to regenerate in AA, since the target organ is damaged, but not destroyed. In summary, we characterized the role of rare variants in AA susceptibility and identified a disease mechanism for hair shaft cuticle integrity in AA pathogenesis.

## Methods

This study complied with all relevant ethical regulations for work with human participants (five institutions local IRB committees, please see below for more detail) and animal subjects (Columbia University’s Institutional Animal Care And Use Committee). The authors affirm that human research participants provided informed consent for publication of the images in Fig. 3a.

### Sequencing sample selection

DNA samples from AA patients were obtained for sequencing from the National Alopecia Areata Registry (NAAR) and the Columbia University local repository. All samples were obtained following patient consent and approval by the respective Institutional Review Boards. The study was approved by the local IRB committees from the five institutions that participate in the National Alopecia Areata Registry, including the University of Texas, MD Anderson Cancer Center; Columbia University Irving Medical Center; University of Minnesota, Minneapolis; University of Colorado, Denver; and University of California, San Francisco. Samples were prioritized on self-reported Caucasian ethnicity and positive family history of disease. A small number of Non-Caucasian samples were included in sequencing, and removed from this analysis using Eigenstrat and principal component analysis. Scalp biopsies used for IHC and expression analysis were collected from four National Alopecia Areata Foundation (NAAF) registry sites and Columbia University Dermatology Department. Institute for Genomic Medicine (IGM) controls were selected from >45,000 whole exome sequenced individuals housed in the IGM Data Repository. To select a population control group (*n* = 18,531) for comparison with the AA patients, we used the database of previously sequenced exomes from unrelated individuals in the Institute of Genomic Medicine (IGM) at Columbia University, which is available for comparison through unrelated studies. We excluded phenotypes potentially related to AA such as infectious disease, primary immune deficiency, and endocrine disorders. This use of the data for control purposes in future studies had been previously consented by participants. We employed principal components analysis (PCA) to identify an appropriate control population, corresponding to the ancestry of AA patients (as described in “Methods”). In total, after quality control process and PCA 18,531 unrelated individuals were included as controls in the collapsing analysis.

### Whole exome sequencing

Whole Exome Sequencing of DNA was performed at Columbia University^7^. Briefly, Agilent All Exon kits (V4 UTR, V5 UTR, and CRE), Nimblegen SeqCap EZ Exome Enrichment kits (V2.0, V3.0, VCRome and MedExome) and IDT Exome Enrichment panel were used for whole exome capture. Sequencing was performed on HiSeq 2500 and NovaSeq sequencers according to standard protocols.

Illumina lane-level FASTQ files were aligned to the Genome Reference Consortium Human Build 37 (GRCh37) using the Burrows-Wheeler Alignment Tool (BWA)^56^ and used the Picard software (http://picard.sourceforge.net) to remove duplicate reads and process lane-level SAM files to create a sample-level BAM file. GATK was used to recalibrate base quality scores, realign indels and call variants^57^. To control and test for potential kit biases, we performed several tests (described in detail below): (1) Population Stratification using a Principal component analysis (2) Sample level quality filters (3) Coverage harmonization between cases and controls and 4) Inflation testing. Using these steps, we remove both cases and controls that are (1) related, (2) bad quality, and (3) genetic outliers either to specific ancestry or kit origin, both of which will cause a bias to the results. The further test of coverage between cases and controls (harmonization) assures than even if samples are not genetic outliers, but have significantly better coverage in one cohort vs. the other in specific positions that might affect variant calling in those positions, these positions will not be taken into account when performing the burden testing. Last, the inflation test of the results is a final test to examine whether the results exhibit gene *p*-value distribution that shows an excess of significant signals, which would be the result of a bias towards one group or the other that is either ancestry or kit specific. However, we observed that the genomic inflation factor (λ) which examines that distribution, especially of the main LoF model, is very close to one (λ = 1.03) suggesting no inflation in the results and confirming that the *KRT82* signal is valid.

### Sample quality and relatedness filters

The initial sample consisted of 873 AA cases and 18,581 controls. Samples reporting >8% contamination according to VerifyBamID^58^ were excluded. KING^59^ was used to ensure only unrelated (up to third-degree) individuals contributed to the analysis. Furthermore, to be eligible for analysis, we required samples to have >87% of the consensus coding sequence (CCDS) covered at 10×.

### Population stratification

Once we controlled for low quality and related samples, we applied an additional analysis to control for population stratification by using EIGENSTRAT^60^ to remove samples that were considered as genetic outliers. This ensured that the main cluster of samples was of similar genetic origins and would not be biased by sub-population that are genotypic outliers.

### Variant-level quality control

Quality thresholds were set based on previous studies^7,8,61^. Variants were required to have a quality score ≥30, quality by depth score ≥2, genotype quality score ≥20, read position rank sum ≥ −3, mapping quality score ≥40, mapping quality rank sum > −10 and a coverage ≥10. SNVs had a maximum Fisher’s strand bias of 60, while indels had a maximum of 200. For heterozygous genotypes, the alternative allele ratio was required to be greater than or equal to 25%. Variants were excluded if they were marked by EVS as being failures^62^. Variants were annotated to Ensembl 73 using SnpEff^63^.

### Variant-level coverage harmonization between cases and controls

To ensure balanced sequencing coverage of evaluated sites between cases and controls, we imposed a statistical test of independence between the case/control status and coverage^7^. Briefly, we tested for this independence by performing a two-sided binomial test on the number of covered samples at given site. We used the binomial test for coverage balance in all the burden analyses described in this study as an additional qualifying criterion. Any site that resulted in a nominal significance threshold of 0.01 or lower was eliminated from further consideration.

### Gene-based collapsing analysis

In this manuscript, we performed gene-based collapsing analyses based on different qualifying criteria for each model. Burden tests were applied to our WES data as a method for aggregating variants within a given gene and comparing the frequency of genetic variants in cases and controls. In a gene-collapsing burden test, variants are annotated as “qualifying variants” based on specified criteria, such as allele frequency in the general population and variant effect. These specified criteria composed our seven different models utilized in this study. Variants that meet the parameters are identified as ‘qualifying’ and are then combined together based on gene boundaries, thereby determining whether a gene harbors more *total rare* damaging variants in cases compared to controls. Our first model was designed to search for rare, loss of function (LOF) variants with a MAF ≤ 1% in cases, internal controls, each population in the gnomAD database^16^. The second model required rare (MAF ≤ 1%), non-synonymous coding variants that were predicted as possibly or probably damaging by PolyPhen‐2 HumVar^17^. The third model presented built upon the LOF and missense models with the inclusion of qualifying high TraP damaging synonymous and splice region variants.

We performed four additional gene-based analyses: Three of the additional models examined variants with different thresholds of population allele frequencies, including a more common MAF of ≤5%, as well as more rare variants with MAF ≤0.1% and ≤ 0.01%. Last, our study included a recessive model that used a 1% MAF threshold and additionally qualified compound-het cases. The four additional models were not included in the main text of this work as they did not identify any candidate genes that passed genome-wide threshold, except the common 5% model. This model identified genes that passed genome and study-wide significance thresholds. However, the model was discarded due to the large inflation (λ = 2.17) that was a result of the inclusion of common variants and subsequent sub-populations.

For each of these seven models, we tested the list of 18,653 CCDS genes. For each gene, we counted the presence of at least one qualifying variant in the gene. A two-tailed Fisher’s exact test (FET) was performed for each gene to compare the rate of cases carrying a qualifying variant compared to the rate in controls. Genome-wide significance threshold was adjusted for multiple testing of 18,653 genes yielding α = 2.68E−06 (0.05/18653). For our study-wide significance threshold, after Bonferroni correction for the number of genes tested across the seven non-synonymous models, the study-wide multiplicity-adjusted significance threshold α = (0.05/ [7*18653]) = 3.83E− 07. We did not correct for the synonymous (negative control) model.

Due to KRT82 being tolerant to LoF variants, we constructed a complementary model to investigate the association of common LOF variants in *KRT82* in cases compared to controls. The complementary LoF model was designed to search for common loss of function (LOF) variants with a MAF > 1% in cases, internal controls and each population in the gnomAD database, considering all 18,653 CCDS genes.

### Conservation annotation

Conservation alignment was performed using NCBI Orthologs for KRT82. Orthologs for *KRT82* are present in species in the Mammalia class. Mammals were selected based on evolutionary close and distant species with and without typical fur coats to understand the scope of evolutionary conservation at this gene locus. Criteria for mammalian classification include the presence of hair, which all selected species (human, mouse, sheep, rhesus monkey, sea lion, vampire bat, and armadillo) fulfill. Protein sequences were aligned and visualized using Boxshade. Variant visualization and conservation were performed using Plot Protein. Chromosome region containing keratin genes was visualized using NCBI Sequence Viewer.

### Immunohistochemistry

Human scalp biopsies were obtained through our AA patient cohort^36^. Briefly, skin punch biopsy specimens were fixed in the PAXgene Tissue Containers and shipped overnight to Columbia University. Samples were bisected, with one half of the sample for tissue embedding and one half for RNA extraction (see below). Samples were formalin-fixed, paraffin embedded, and sectioned at 10 microns. Slides were deparaffinized and rehydrated, followed by citrate antigen retrieval (VECTOR cat# H-3300-250) for 15 min in a pressure cooker. Slides were washed with PBS and H_2_0, and the peroxidase was quenched with 3% H_2_O_2_ for 10 min at RT, followed by PBS washes. Samples were blocked for 30 min at RT with goat serum and incubated overnight with either 1:100 CD8 primary antibody (Abcam Cat# ab101500) or KRT82 primary antibody (Progen cat# GP-HHB2). After PBS washes, slides were incubated with either; and followed by PBS washes. Vectastain Elite ABC-HRP kit (VECTOR cat#PK-6200) was added to samples for 30 min, followed by incubation with DAB-substrate (VECTOR SK-4100) and DI H20 to stop the reaction. Nuclei were stained with Gill’s Hematoxylin and samples were dehydrated and mounted.

### Animals

C57BL/6J mice were bred and maintained in a pathogen-free animal facility in the Russ Berrie Medical Sciences Pavilion in accordance with guidelines provided by the Institute of Comparative Medicine and Institutional Animal Care and Use Committee at Columbia University. Mice were socially housed under a 12-h light/dark cycle. All mice were bred in the facility and therefore all birth dates and ages were accurately documented.

### RNA Expression

All RNA expression studies utilized samples from our AA patient cohort^36^. One half of the bisected sample was processed using the PAXgene tissue miRNA kit to extract RNA. Library prep was performed for microarray analysis using Ovation RNA Amplification System V2 and Biotin Encore kits (NuGen Technologies, Inc., San Carlos, CA). Samples were subsequently hybridized to Human Genome U133 Plus 2.0 chips (Affymetrix, Santa Clara, CA) and scanned at the Columbia University Pathology Core or the Yale Center for Genome Analysis. Quality control of microarrays was performed using the affyAnalysisQC package from http://arrayanalysis.org/. Microarray preprocessing was performed using BioConductor in R. The dataset was normalized using GCRMA and MAS5. The Affymetrix HGU-133Plus2 array contains 54675 probe sets (PSIDs). Filtering was performed so that PSIDs that were on the X or Y chromosome, that were Affymetrix control probe sets, or that did not have Gene Symbol annotation were removed from all arrays for further downstream analysis. Correction for batch effects was performed using the implementation of the function ComBat available in the sva package with gender and AA group (AT/AU, AAP, and normal) used as covariates.

The ALADIN heatmap was constructed using the RNA expression data based on the inflammatory gene expression signatures (CTL and IFN) previously established (PRF1, CD8A, GZMB, ICOS, STAT1, MX1, CXCL9, CXCL10, CXCL11)^64^.

### KRT82 Expression analysis

Differential analysis was performed using the RNA expression data in 98 individuals (36 controls, 30 AAP, 32 ATAU). A subset of AAP patients provided biopsies from both non-lesional and lesional scalp areas providing 122 datapoints for differential expression analysis. Significance was determined using an unpaired t-test with Welch’s correction. Integration of patient expression and sequencing data was performed on 32 individuals with multi-dimensional data available (12 controls and 20 AAP patients). None of the 12 controls had a qualifying *KRT82* variant, and 2 AAP patients had a rare, damaging variant, for whom we had both lesional and non-lesional biopsies for analysis. Expression data is displayed in boxplots in which each sample data point is represented by individual dots; the center line denotes the median; upper and lower limits of the box denote 75th and 25th percentiles, respectively; and the whiskers indicate the dataset maximum and minimum.

### Haplotype analysis

SHAPEIT v2.17 was used for phasing and haplotype construction of the 15 heterozygous AA individuals with the Arg47X variant (12-52799923-G-A). Briefly, the genotypes of all SNPS within the KRT82 gene domain were extracted for the 15 individuals. Multiallelic variants and variants that were missing data in more than 20% of samples were removed, resulting in 83 variants that remained for haplotype construction. SHAPEIT developers’ recommendation suggested removing any samples with missing genotypes in more than 5% of variants; however, we overrode this recommendation in an effort to analyze all 15 AA patient haplotypes. After phasing, shared haplotypes were manually assigned based on SNPs that had a variant in at least one individual (n = 21 variants). Four individuals with missing genotypes resulted in inconclusive haplotype assignment, and as a result, 13 phased haplotypes were manually constructed, with 7 different haplotypes carrying the Arg47X variant. DNA Methylation was visualized using UCSC Genome Browser.

### Immunofluorescent Imaging

Dorsal mouse skin was dissected from C57BL/6J male mice at telogen (p22, p90), anagen (p31), and catagen (p43) phases of the HF. For human sample biopsies, control subjects and moderate to severe AA participants were selected and voluntary informed consent was obtained. Biopsies were bisected immediately after sampling at the bedside and placed in sterile water for analysis. All Institutional protocols, IRB standards and International council on harmonization (ICH) and Good Clinical Practice (GCP) standards were upheld. Mouse and human skin samples were embedded in clear Tissue Plus Optimal Cutting Temperature (OCT) compound (Fisher HealthCare cat# 23-730-571) and flash frozen in liquid nitrogen. Samples were sectioned at 10 microns using Leica CM1850 UV Cryostat. Slides were fixed in 4% paraformaldehyde (PFA) for 10 min and washed with PBS. 5-min Permeabilization with 0.1% Triton in PBS was followed by 1-h block in 5% goat serum/0.1% Triton solution. KRT82 (Progen cat# GP-HHB2) primary antibody was incubated at 1:200 in blocking solution overnight at 4 °C. Slides were washed in PBS and incubated with anti-guinea pig secondary antibodies (Invitrogen cat# A-11073) at 1:1000 in blocking solution for 1 h at room temperature. Samples were again washed in PBS, mounted in Fluoroshield with DAPI (Sigma cat# F6057) mounting media, and imaged on Zeiss LSM 5 Exciter confocal microscope. Images were processed using ImageJ32 to make color composite images, merge green and blue channels, and make maximum intensity z-projections. In photoshop, overlapping images were stitched together using Photomerge and identical color channel adjustments were applied to all images and samples.

### Immunoblotting

Protein was harvested from the dorsal skin of C57BL/6J male mice at various stages of the hair cycle. Samples were lysed with RIPA Lysis Buffer + 1% protease inhibitor/1% phosphatase inhibitor (Sigma cat# R0278, cat# P8340, cat# P2850). Lysates were agitated at 4 °C for 30 min prior to centrifugation (16,000 × *g*, 20 min at 4 °C) and collection of supernatant. Sample concentration was determined using the Bradford Assay. Equal amounts of protein were then loaded onto polyacrylamide gels for electrophoresis, transferred onto methanol-activated PVDF membranes, and blocked with 5% milk in TBST. Blots were incubated with KRT82 primary antibody (Progen cat# GP-HHB2) at 1:2000 overnight at 4 °C, and then HRP-conjugated anti-guinea pig IgG secondary antibody (Jackson ImmunoResearch cat# 106-035-033) at 1:10,000 at RT. To probe for loading controls, HRP-conjugated b-actin antibody (Santa Cruz cat# sc-47778) was incubated at 1:5000 for 45 min at RT. Immobilon Western Chemiluminescent HRP substrate (Millipore cat# WBKLS0500) was used for detection and imaged using the Bio-Rad ChemiDoc MP Imaging System.

### Reporting summary

Further information on research design is available in the Nature Research Reporting Summary linked to this article.

## Supplementary information

Supplementary Information

Reporting summary

## Data Availability

Exome sequencing data of *KRT82* that support the findings in this study have been deposited in dbGaP repository with accession codes phs002632.v1.p1. The raw exome sequencing BAM files submitted in Sequence Read Archive(SRA) under accession number PRJNA768921. The processed phenotypic files of human exome data are available at dbGaP (https://www.ncbi.nlm.nih.gov/projects/gap/cgi-bin/study.cgi?study_id=phs002632.v1.p1). Users can gain access by following the dbGaP data access protocol explained here: https://www.ncbi.nlm.nih.gov/projects/gapprev/gap/cgi-bin/GetPdf.cgi?document_name=GeneralAAInstructions.pdf. Full immunoblots for Fig. 6d are provided in the Source data file. Source data are provided with this paper.

## Source data

Source data

## Code availability

The code used in this manuscript for gene-level burden analysis of the exome data was previously published in Gelfman et al.^18^. The ATAV software was constructed by the Institute for Genomic Medicine and the code is published online at https://github.com/nickzren/atav.
