# Supplementary material for: Whole exome sequencing in Alopecia Areata identifies rare variants in KRT82
Source: Nat Commun. 2022 Feb 10;13:800. doi: 10.1038/s41467-022-28343-3 (PMC8831607; doi:10.1038/s41467-022-28343-3)
Supplement: Supplementary file 1 — Supplementary Information [file 41467_2022_28343_MOESM1_ESM.pdf]

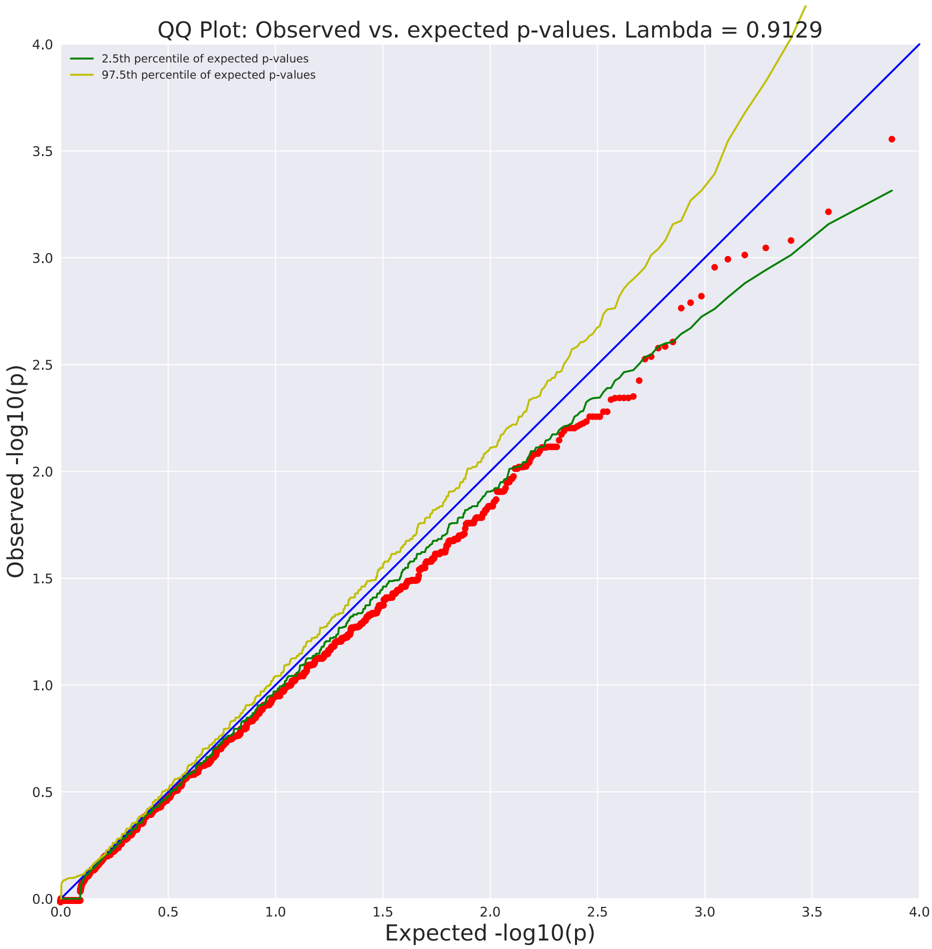

Supplementary Fig. 1 Rare Synonymous Variant-Gene Collapsing Model. Qualifying variants in this model included any rare (MAF ≤ 0.01%) and synonymous variants, that are predicted to have no pathogenic role. Low inflation (lambda (λ) = 0.91) of representative Q-Q plot. No genes reached genome-wide significance in the rare synonymous variant model, serving as a negative control. Synonymous variants do no affect the protein product, and therefore should not have a pathogenic effect or be associated with disease. Under this rationale, distribution of p-values should follow the null distribution without any significant associations, as seen here.

Model	Gene	Cases	Controls	Case Frequency	Control Frequency	P-value
Synonymous	AQP2	6	13	7.1%	0.1%	2.79E-04
	OTOF	0	151	0%	1.0%	6.09E-04
	HS1BP3	7	24	0.8%	0.2%	8.30E-04

Supplementary Table 1 No genes in the rare synonymous variant model (negative control model) reach genome-wide significance p-value was determined by two-tailed Fisher's exact test (FET).

Haplotype ID	12-52788928-G-A	12-52789140-T-C	12-52789198-C-A	12-52789208-T-C	12-52789509-T-C	12-52789597-A-G	12-52789605-T-A	12-52789691-A-C	12-52789693-GT-G	12-52790194-C-A	12-52790385-T-C	12-52790816-C-T	12-52790899-T-C	12-52790940-C-T	12-52794642-A-G	12-52795351-C-A	12-52797617-G-A	12-52797862-T-C	12-52799341-C-T	12-52799419-T-C	12-52799923-G-A
Hap01	A	C	A	C	C	G	A	C	G	A	C	T	C	T	G	A	A	C	T	C	G
Hap02	A	C	A	C	C	G	A	C	G	A	C	T	C	T	G	A	A	C	C	C	G
Hap03	G	T	C	T	T	A	A	A	GT	A	T	T	T	C	A	A	A	C	C	T	G
Hap04	A	C	A	C	C	G	A	C	G	C	C	T	C	T	G	A	A	C	T	C	G
Hap05	A	C	C	T	C	G	A	C	G	A	C	T	C	T	G	A	A	T	T	T	G
Hap06	A	C	A	C	C	G	A	C	G	A	T	T	T	T	A	C	A	C	T	C	G
Hap07	A	C	A	C	C	G	A	C	G	A	C	C	C	T	G	A	A	C	T	C	G
Hap08	A	C	A	C	C	G	**A**	C	G	A	C	T	C	T	G	A	A	C	T	C	A
Hap09	G	T	C	T	C	G	A	A	GT	A	C	T	C	C	G	A	**G**	C	C	T	A
Hap10	A	C	A	C	C	G	**T**	C	G	A	C	T	C	T	G	A	A	C	T	C	A
Hap11	G	T	C	T	C	G	A	**C**	**G**	A	C	T	C	C	G	A	A	C	C	T	A
Hap12	G	T	C	T	**T**	**A**	**T**	A	GT	A	C	T	C	C	G	A	A	C	C	T	A
Hap13	G	T	C	T	C	G	A	A	GT	A	C	T	C	C	G	A	A	C	C	T	A

Supplementary Table 2 Arg47X alternate allele (A) resides on at least 6 distinct haplotypes. Each column represents a variant, and each row represents a haplotype. Arg47X alternate allele (A) that results in a premature stop codon is highlighted in red in the last column. After phasing, we report that the A allele resides on at least six distinct haplotypes. In this region, thirteen total haplotypes are observed. Black bolded boxes denote where the haplotypes differ..
